# Supplementary material for: CRISPR/Cas9-Mediated Targeting of BPV-1-Transformed Primary Equine Sarcoid Fibroblasts
Source: Viruses. 2023 Sep 17;15(9):1942. doi: 10.3390/v15091942 (PMC10536948; doi:10.3390/v15091942)
Supplement: Supplementary file 1 [file viruses-15-01942-s001.zip › viruses-2588587-supplementary.pdf]

Supplementary Table S1:

Primary fibroblast culture	Age (years)	Species	Breed	Gender	Sarcoid type	Location	Multiple sarcoids	Age (years)
EqS#1	15	Horse	Swiss Warmblood	Mare	Fibroblastic	Inguinal	Yes	15
EqS#4	12	Horse	Quarter Horse	Gelding	Nodular	Prepuce	Yes	12
EqS#5	6	Horse	Swiss Warmblood	Stallion	Fibroblastic	Ear	Yes	6
EqS#6	5	Horse	Irish Cob	Mare	Nodular	Inguinal	Yes	5
Ctrl Fbs	30	Pony	Minishetty	Gelding	-	Inguinal	-	30

Supplementary Table S1. Clinical data of the horses included in the study.

Supplementary Table S2:

**BPV-1 PCR:**							
Frag-ment	Primer fwd (5′-3′)	Primer rev (5′-3′)	Ref.	Denatura-tion	Annealing	Elong-ation	Fragment size (bp)
Abl-1	CAAAGGCCAGT AGCATCTG	GTAGCCTAAGAC CCGGAGC	modified from (1)	95°C, 45s	59°C, 45s	72°C, 1min	798
E2	AGAACACACTG CTTTATGC	CAAACTCCACCT CTACCAC	1	95°C, 45s	59°C, 45s	72°C, 1min	248
E5	CACTGCCAT TGCCTTTTC	GGAGCACTCAA AATGATCCC	1	95°C, 45s	59°C, 45s	72°C, 1min	105
E6	GCTGAATTATTG CATGGCAAAA	CTATGGGTATTT GGACCTTGAA	1	95°C, 45s	59°C, 45s	72°C, 1min	141
E7	CGTTGCTGATTTT AAGTCCAT GTG	GGGACCTCGCTT CCTAGTAGGA	2	95°C, 45s	59°C, 45s	72°C, 1min	116
L1	TATCGCTATATA GAGTCTCCTG	GACACAGCACA TTGCTATTAAG	1	95°C, 45s	59°C, 45s	72°C, 1min	344
E2 seq	GATGAATCGGGT GAGCAAC	CAAACTCCACCT CTACCAC		95°C, 45s	58°C, 45s	72°C, 1min	476
E5 seq	CAAAGGCAAGA CTTTCTGAAAC	GACCTGTACAGG AGCACTCAA		95°C, 45s	63°C, 45s	72°C, 1min	255
**TIDE PCR**							
Vim E1	AGCTGCAGGAG CTGAATGAC	GCTGCAGTCCCG ACACAT		98°C, 7s	64°C, 20s	72°C, 20s	516
LCR	CATTACTGGTGC ATAGCGGATG	GACAGTCCAACC CTGAGAATG		98°C, 7s	63°C, 30s	72°C, 20s	495
E5	CTGATAACGGTG CTGAAAGACAA G	CTTGCATGTCCT GTACAGGTC		98°C, 7s	64°C, 20s	72°C, 20s	549
E6	CTCACCGAAACC GGTAAGTAAG	CTATGGGTATTT GGACCTTGAA		98°C, 7s	64°C, 20s	72°C, 20s	558

Supplementary Table S2. Overview primers and PCR conditions. For the BPV-1 PCR the GoTaq G2 Green MasterMix was used and the initial denaturation was performed at 95°C for 2 min. TIDE PCR were run using the Phusion High-Fidelity PCR Master Mix and the initial denaturation condition was 98°C for 30 sec. For both master mixes, final elongation was performed at 72°C for 6 min and 35 cycles were run.

References for Primers:

1) Nasir L, Reid SW. Bovine papillomaviral gene expression in equine sarcoid tumours. Virus Res. 1999 Jun;61(2):171-5. doi: 10.1016/s0168-1702(99)00022-2. PMID: 10475087.
2) Yuan, Z., Philbey, A. W., Gault, E. A., Campo, M. S., & Nasir, L. (2007). Detection of bovine papillomavirus type 1 genomes and viral gene expression in equine inflammatory skin conditions. Virus research, 124(1-2), 245–249. https://doi.org/10.1016/j.virusres.2006.10.012

<u>Supplementary Figure S1:</u>

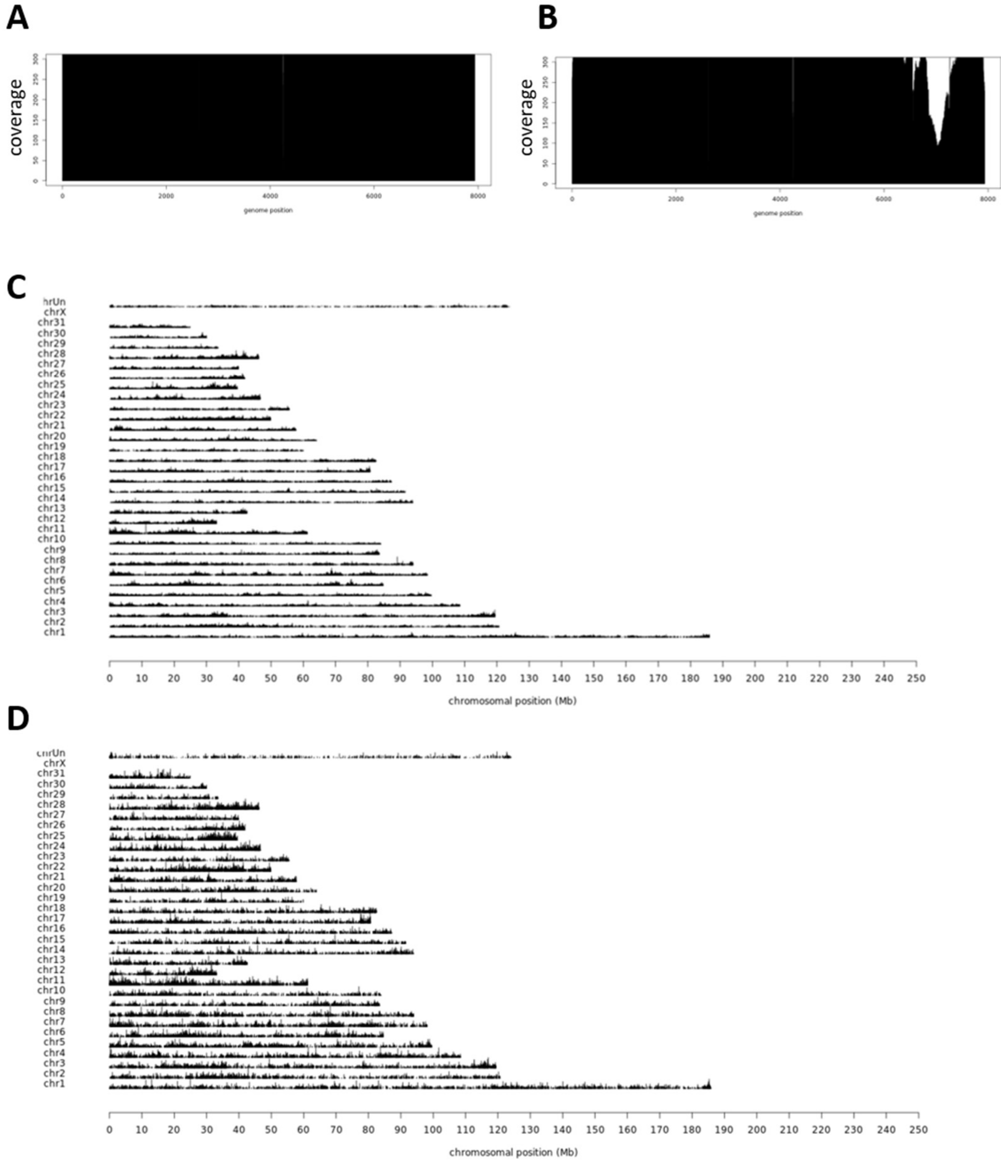

Supplementary Figure S1: Example of a TLA coverage plot derived from an equine sarcoid and mapped across the BPV-1 sequence (A, B) and across the horse genome (ecuCab2, C, D). (A, B) The y-axis depicts the coverage; the x-axis shows the position within the BPV-1 sequence. The results obtained using primer set 1 (BPV-1-E1) are shown in panel A, those with primer set 2 (BPV-2-E7) in panel B. (C, D) The y-axis depicts the coverage, the x-axis depicts the chromosomal position. The results obtained using primer set 1 are shown in panel C, those with primer set 2 in panel D.

Supplementary Figure S2:

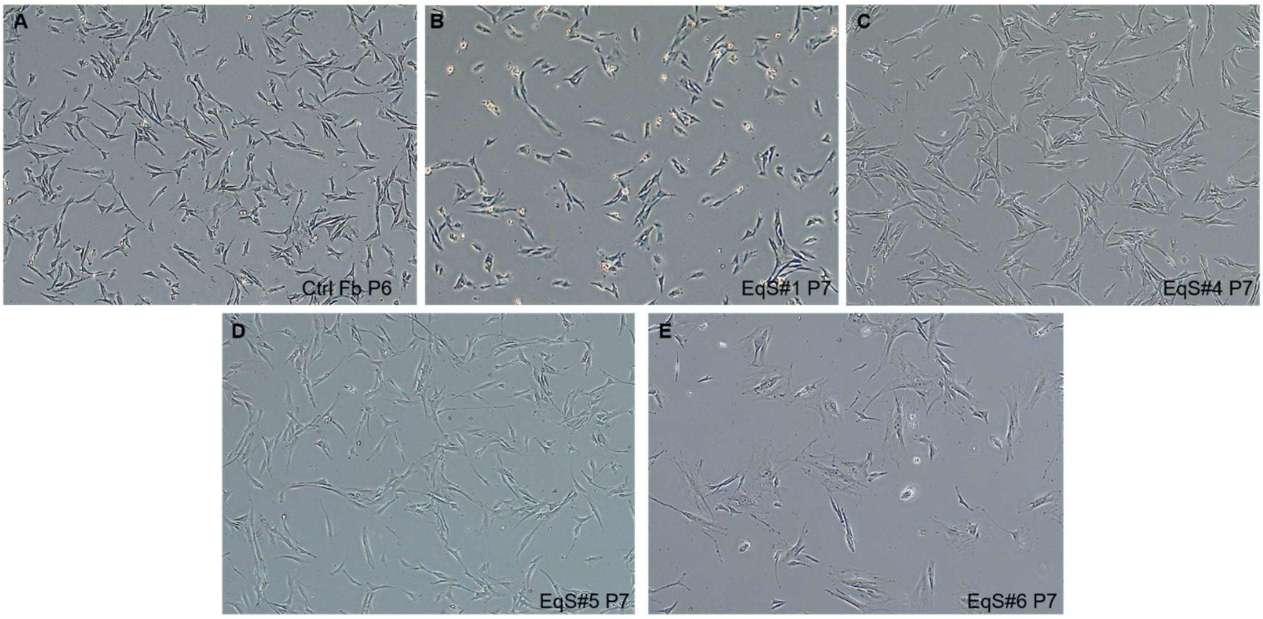

Supplementary Figure S2: Morphology of Ctrl and EqS fibroblasts. (A) Ctrl Fbs, (B) EqS#1, (C) EqS#4, (D) EqS#5 and (E) EqS#6 cultures display an elongated cell body with fine processes extending towards neighboring cells.

Supplementary Figure S3:

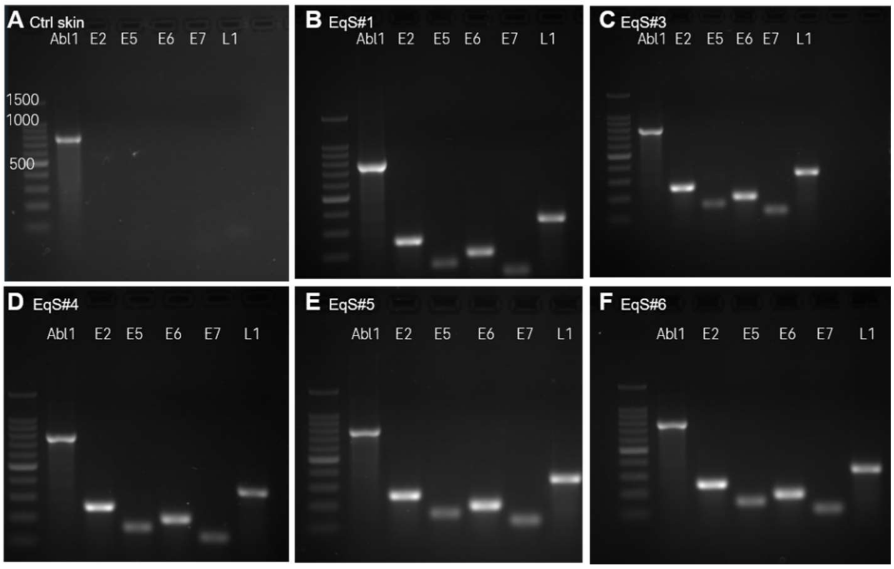

Supplementary Figure S3: PCR to assess the DNA of BPV1 oncogenes in control dermis and EqS tumors. E2, E5, E6, E7 and L1 were not detected in the control dermis sample (A). The EqS#1 (B), EqS#3 (C), EqS#4 (D), EqS#5 (E), and EqS#6 (F) tumor tissue showed specific bands for BPV oncogenes. Equid ABL1 PCR was performed to confirm PCR compatibility of all DNA isolates. Ladder: Promega BenchTop 100bp DNA Ladder; G8291.

Supplementary Figure S4:

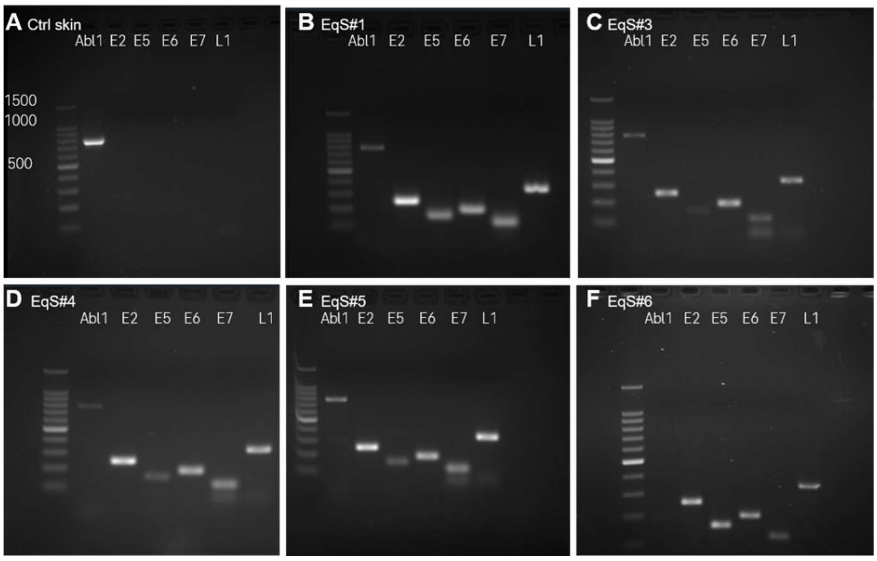

Supplementary Figure S4: RtPCR to assess the mRNA of BPV1 oncogenes in control dermis and EqS tumors. E2, E5, E6, E7 and L1 were not detected in the control dermis sample (A). The EqS#1 (B), EqS#3 (C), EqS#4 (D), EqS#5 (E), and EqS#6 (F) tumor tissue showed specific bands for BPV oncogenes. Equid ABL1 rtPCR was performed to confirm PCR compatibility of the cDNA. Ladder: Promega BenchTop 100bp DNA Ladder; G8291.

Supplementary Figure S5:

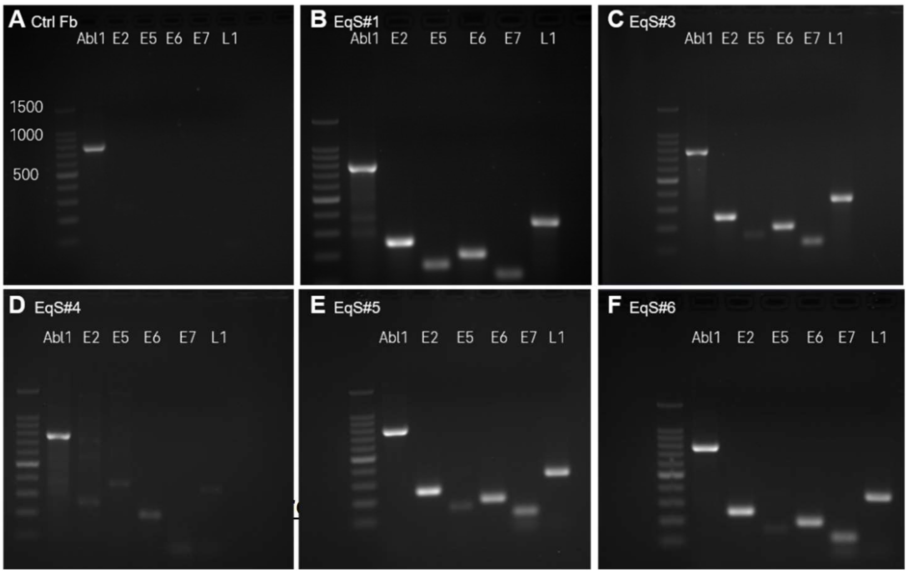

Supplementary Figure S5: PCR to assess the DNA of BPV1 oncogenes in ctrl Fbs and EqS cells. E2, E5, E6, E7 and L1 were not detected in the Ctrl Fbs (A). The EqS#1 (B), EqS#3 (C), EqS#4 (D), EqS#5 (E), and EqS#6 (F) tumor tissue showed specific bands for BPV oncogenes. Equid ABL1 PCR was performed to confirm PCR compatibility of all DNA isolates. Ladder: Promega BenchTop 100bp DNA Ladder; G8291.

Supplementary Figure S6:

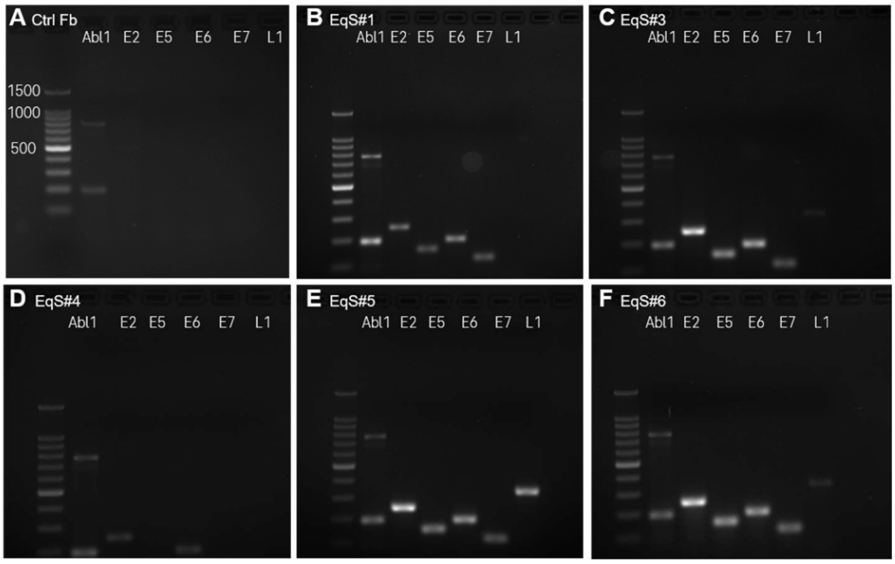

Supplementary Figure S6: rtPCR to assess the mRNA of BPV1 oncogenes in ctrl Fbs and EqS cells. E2, E5, E6, E7 and L1 were not detected in the Ctrl Fbs (A). The EqS#1 (B), EqS#3 (C), EqS#4 (D), EqS#5 (E), and EqS#6 (F) tumor tissue showed specific bands for BPV oncogenes. Of note, no band for L1 was detected in EqS#1 and EqS#4. Equid ABL1 PCR was performed to confirm PCR compatibility of the cDNA. Ladder: Promega BenchTop 100bp DNA Ladder; G8291.

Supplementary Figure S7:

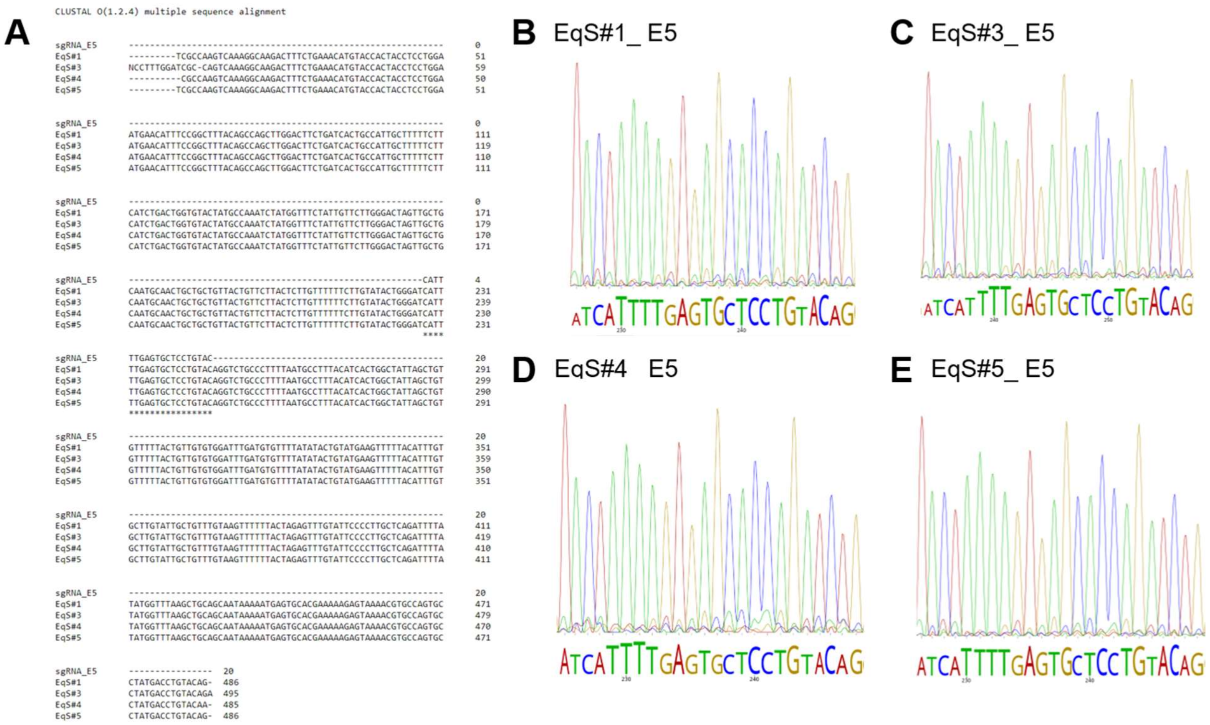

Supplementary Figure S7: Sequencing of E5 DNA from EqS tumor tissue to assess the sgE5 target. (A) Alignment of the E5 sequences of EqS#1, 3, 4 and 5 confirmed sequence homology for the sgE5 target. (B-E) Chromatograms did not indicate point mutations in the sgE5 target locus.

Supplementary Figure S8:

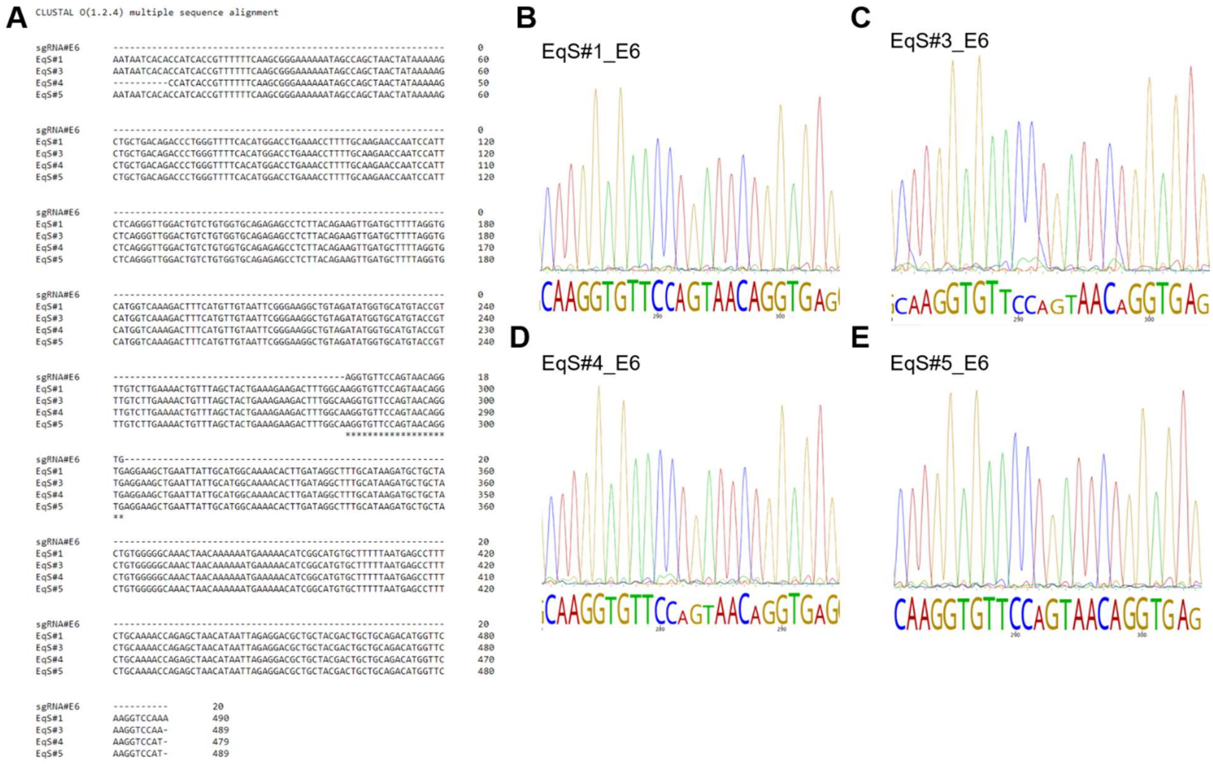

Supplementary Figure S8: Sequencing of E6 DNA from EqS tumor tissue to assess the sgE6 target. (A) Alignment of the E6 sequences of EqS#1, 3, 4 and 5 confirmed sequence homology for the sgE6 target. (B-E) Chromatograms did not indicate point mutations in the sgE6 target locus.

Supplementary Figure S9:

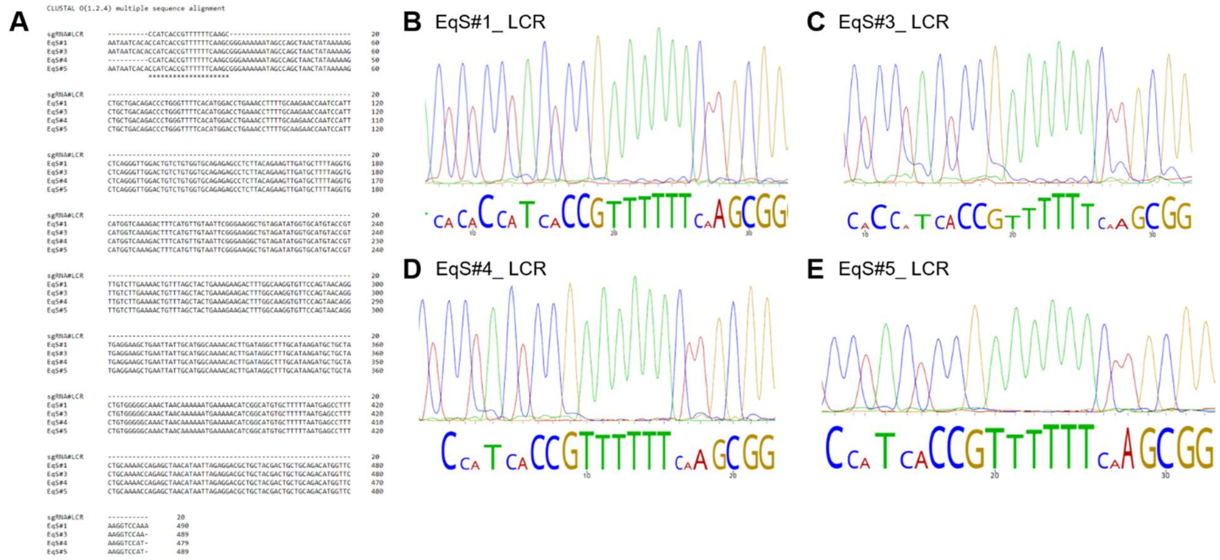

Supplementary Figure S9: Sequencing of LCR DNA from EqS tumor tissue to assess the sgLCR target. (A) Alignment of the LCR sequences of EqS#1, 3, 4 and 5 confirmed sequence homology for the sgLCR target. (B-E) Chromatograms did not indicate point mutations in the sgLCR target locus.

Supplementary Figure S10:

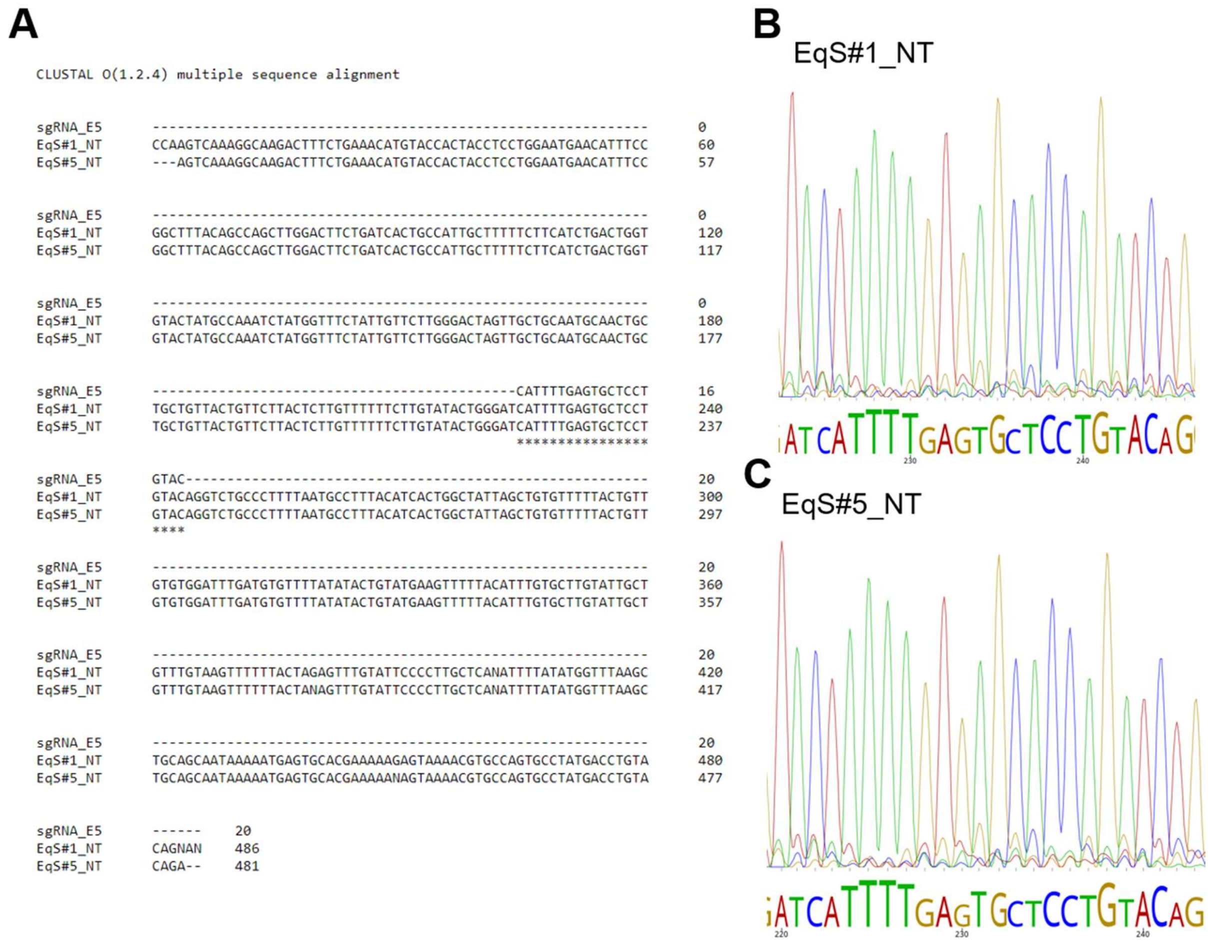

Supplementary Figure S10: Sequencing of E5 DNA from EqS#1 and EqS#5 fibroblasts transduced with NT-gRNA to assess the sgE5 target. (A) Alignment of the E5 sequences of EqS#1-NT and EqS#1-NT confirmed sequence homology for the sgE5 target. (B, C) Chromatograms did not indicate point mutations in the sgE5 target locus.

Supplementary Figure S11:

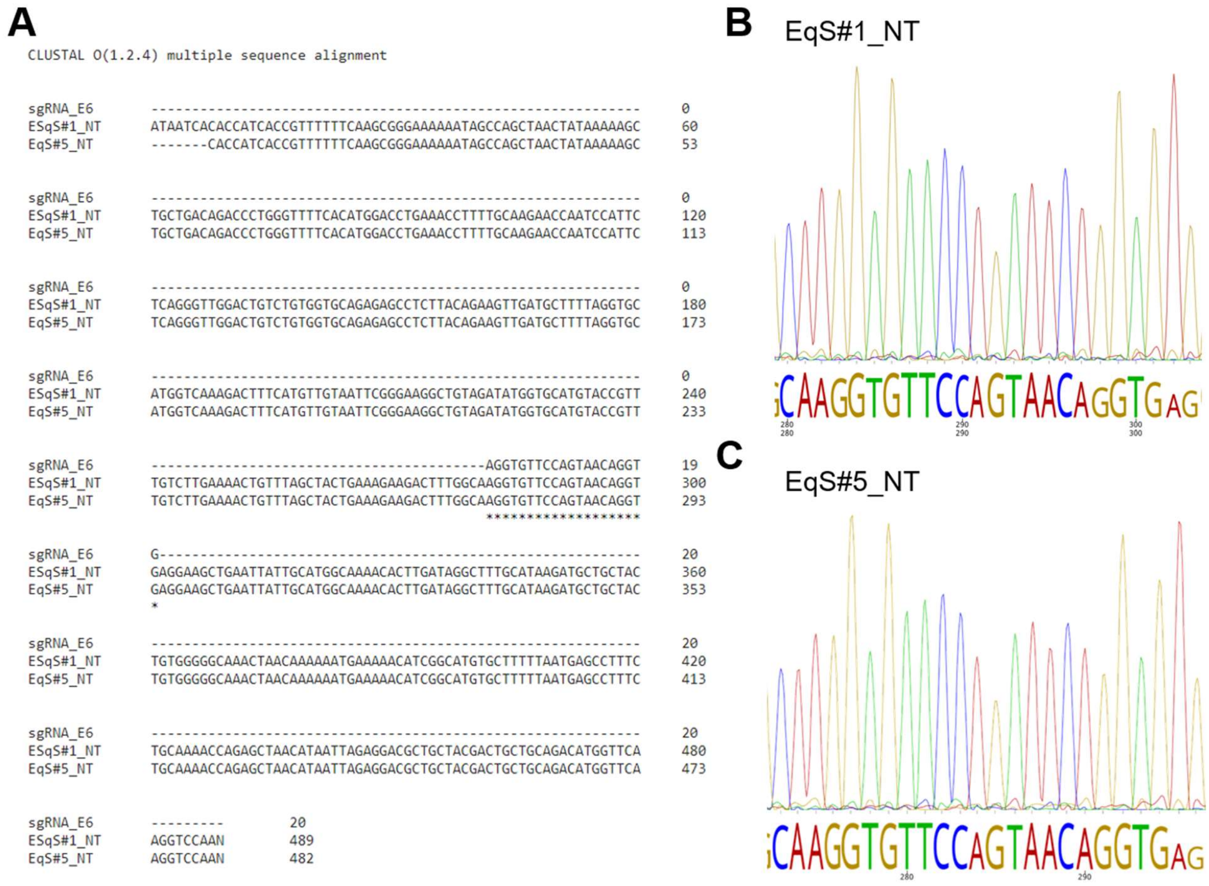

Supplementary Figure S11: Sequencing of E6 DNA from EqS#1 and EqS#5 fibroblasts transduced with NT-gRNA to assess the sgE6 target. (A) Alignment of the E6 sequences of EqS#1-NT and EqS#1-NT confirmed sequence homology for the sgE6 target. (B, C) Chromatograms did not indicate point mutations in the sgE6 target locus.

<u>Supplementary Figure S12:</u>

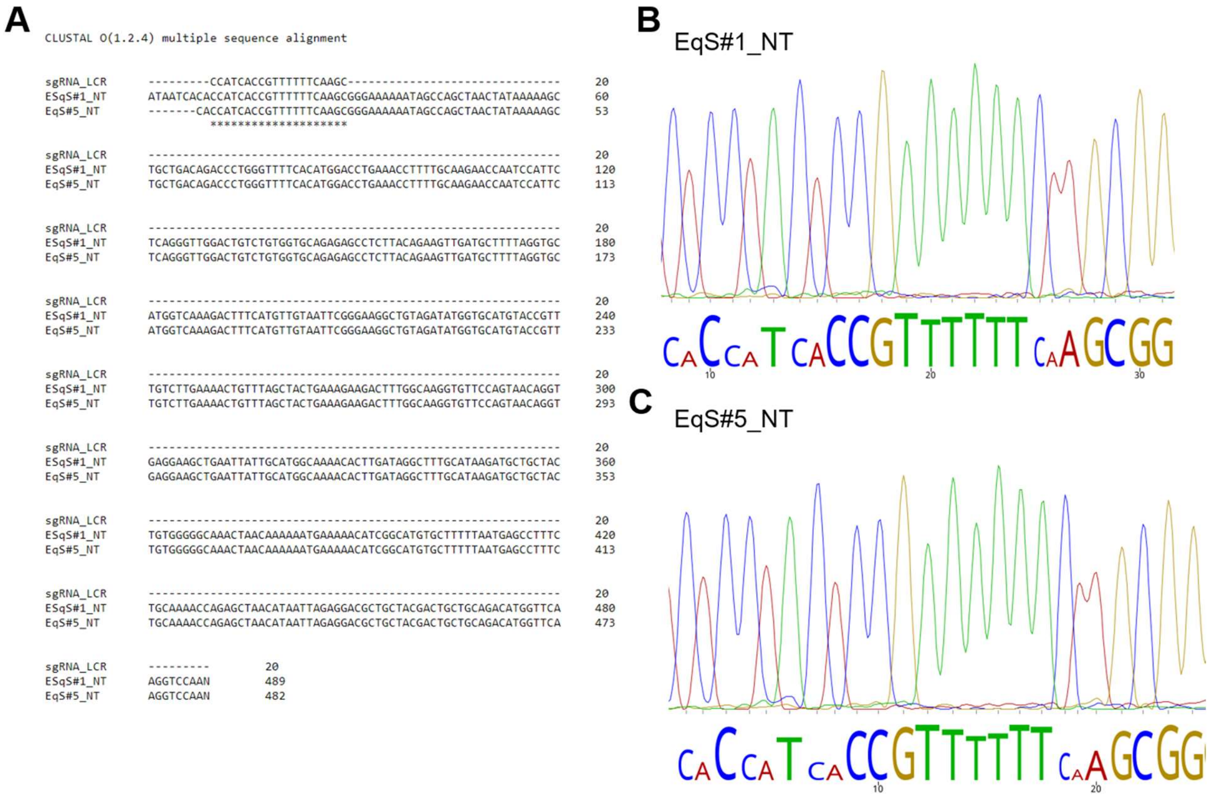

Supplementary Figure S12: Sequencing of LCR DNA from EqS#1 and EqS#5 fibroblasts transduced with NT-gRNA to assess the sgLCR target. (A) Alignment of the LCR sequences of EqS#1-NT and EqS#1-NT confirmed sequence homology for the sgLCR target. (B, C) Chromatograms did not indicate point mutations in the sgLCR target locus.

Supplementary Figure S13:

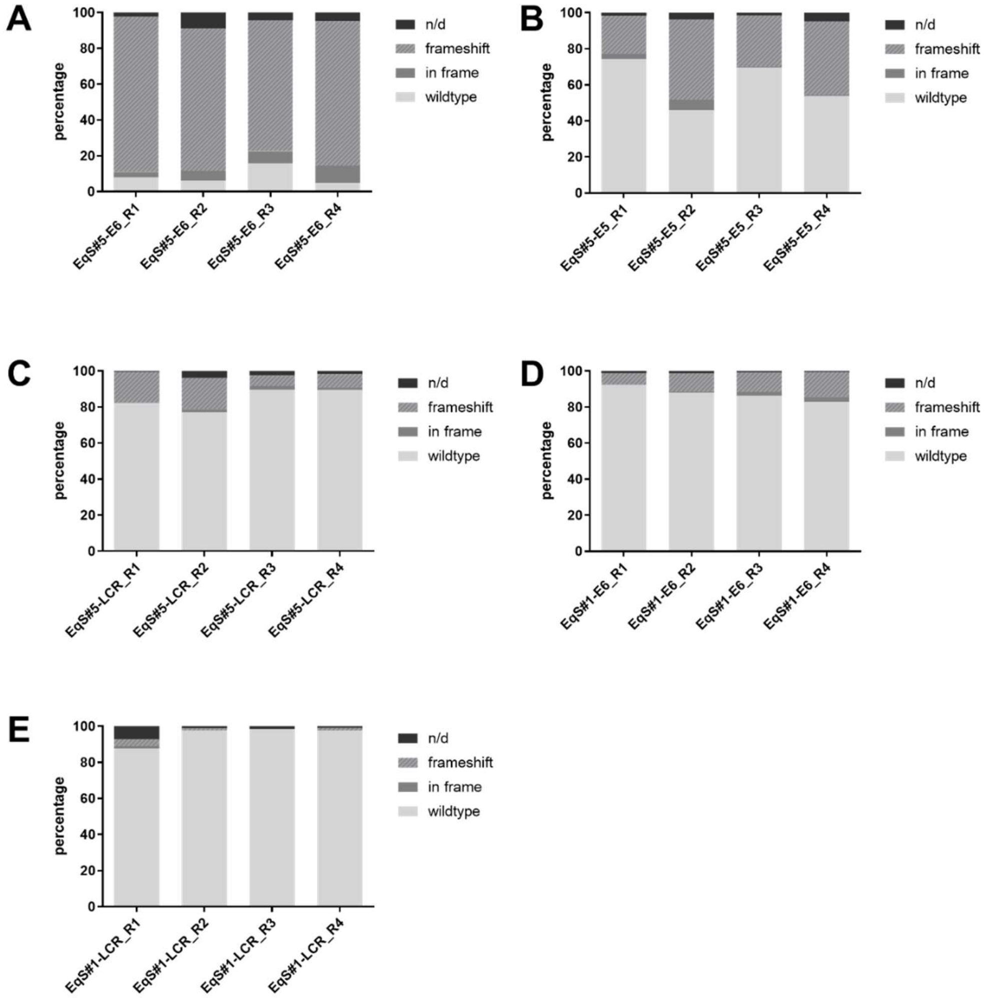

Supplementary Figure S13: Contingency tables of the TIDE analysis of the EqS#1 and EqS#5 cells transduced with BPV-1 specific sgRNAs. (A) TIDE analysis of biological replicates of EqS#5-E6 revealed 73-87% frameshift mutations. (B) The percentage of frameshift mutations in EqS#5-sgE5 varied between 21 and 44%. (C) In EqS#5-sgLCR, the percentage of frameshift mutations was less than 15%. (C) The percentage of frameshift mutations in EqS#5-sgLCR was low and varied between 6 and 17%. (D) In EqS#1-E6 the percentage of frameshift mutations ranged from 6-14%. (E) In EqS#5-sgLCR, the percentage of frameshift was 0-8%. EqS#1, EqS#5: equine sarcoid derived cells; n/d: replacement of nucleotides without frameshift; R: replicate.

Supplementary Figure S14:

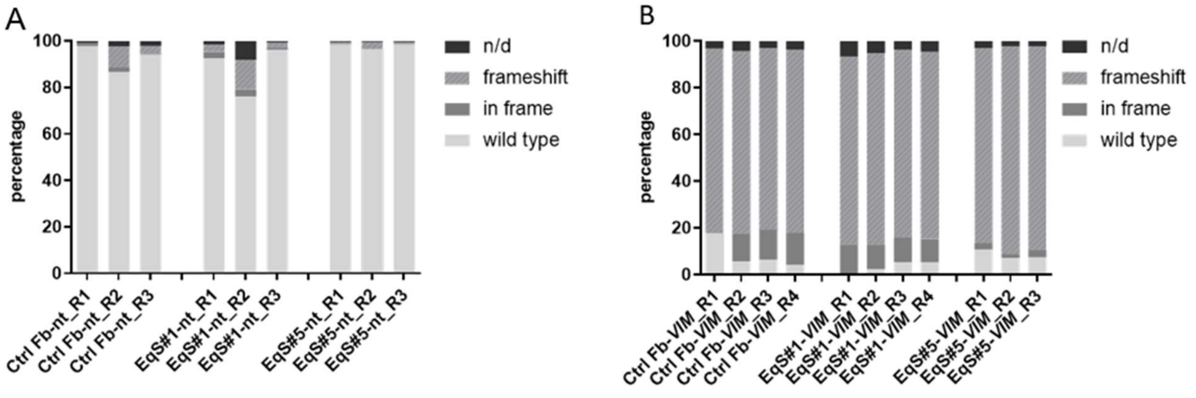

Supplementary Figure S14: Contingency tables of the TIDE analysis of the ctrl Fbs and EqS- cells. (A) TIDE analysis of biological replicates of ctrl Fbs, EqS#1 and EqS#5 transduced with NT-sgRNA. TIDE analysis revealed a low number of frameshift mutations in EqS cells transduced with the NT-gRNA. The higher percentage of frameshift mutations in EqS#1-R2 is most likely related to sequencing errors. (B) TIDE analysis of biological replicates of ctrl Fb, EqS#1 and EqS#5 transduced with *VIM*-sgRNA. TIDE analysis revealed a high percentage of frameshift mutations in *VIM* across EqS cultures with minor variations between EqS cultures and replicates. Ctrl Fb = BPV-1 negative control fibroblasts; EqS#1, EqS#5: equine sarcoid derived fibroblasts; n/d: replacement of nucleotides without frameshift; NT: non-targeting sgRNA; *VIM*: *Vimentin* exon 2-targeting sgRNA.
